# Effect of *Spinacia oleracea* on In Vitro Disintegration and Dissolution of Clopidogrel Bisulfate Tablets

**DOI:** 10.1002/prp2.70223

**Published:** 2026-02-13

**Authors:** Dana Sadaqa, Hani A. Naseef, Asma Radwan, Abdullah K. Rabba, Ramzi H. M. Muqedi

**Affiliations:** ^1^ Pharmacy Department, Faculty of Pharmacy, Nursing and Health Professions Birzeit University Birzeit Palestine; ^2^ College of Pharmacy An‐Najah National University Nablus Palestine

**Keywords:** clopidogrel, dissolution, drug‐food interaction, food effect, spinach, *Spinacia oleracea*, viscosity

## Abstract

Drug–food interactions may compromise therapeutic efficacy, particularly for life‐saving medications such as anticoagulants. Changes in gastric fluid properties, including pH modification and surface film formation, can alter drug dissolution and release. This study evaluated a potential interaction between clopidogrel and spinach and explored the underlying mechanisms. In vitro disintegration and dissolution studies were conducted using HCl and phosphate buffers with and without 5% and 7.5% spinach extract. Drug release was quantified by high‐performance liquid chromatography (HPLC), with six media conditions analyzed in triplicate. Disintegration testing was performed in simulated gastric and intestinal fluids and in the presence of spinach leaves to assess the effect of film formation on tablet wetting and disintegration. In vitro–in vivo extrapolation (IVIVE) was assessed using GastroPlus software. Clopidogrel dissolution decreased with increasing spinach concentration in both media. In HCl buffer, dissolution declined from 99% to 94% and 89%, while in phosphate buffer it decreased from 71% to 66% and 56%. These effects were associated with increased pH (HCl: 1.91, 2.83, 2.98; phosphate: 6.81, 8.83, 6.84), increased solution viscosity, 17.63 to 17.67 and 17.70 in HCl buffer, and from 17.44 to 17.50 and 17.54 in phosphate buffer. Moreover, reduced fluid penetration was due to spinach leaves coverage. IVIVE analysis showed weak correlations (*R* ^2^ = 0.74 in HCl and 0.69 in phosphate buffer). Spinach reduced clopidogrel dissolution in vitro, with statistically significant effects at 5% spinach in HCl buffer (ANOVA test, *p*‐value 0.019) and 7.5% in phosphate buffer (ANOVA test, *p*‐value < 0.001). This interaction appears to be mediated by pH alteration, physical film formation, and potentially metal–drug complexation. Confirmation through in vivo studies is warranted.

## 1 Introduction

Gastrointestinal (GI) absorption of orally administered dosage forms could be influenced by food consumption; in other words, food could affect the GI environment and GI functions such as gastric emptying, intestinal transit time, bile acid secretion, stomach pH, as well as first‐pass effect and liver metabolism [1, 2]. Administering a drug with food is sometimes desirable, where a high‐fat meal is expected to increase drug bioavailability of Class II drugs and will decrease the irritating effect of some drugs such as NSAIDs. When some drugs are co‐administered with certain foods, drug‐food interaction can develop, which further leads to decreased efficacy of the medication by decreasing its release or preventing its excretion, leading to accumulation of the drug in the body, which could possibly lead to toxicity. One of the most well‐known interactions is related to CYP450 3A4 liver enzyme [3]. For example, grapefruit juice consumption should be avoided in case of clopidogrel administration because grapefruit is a strong inhibitor of CYP450 enzyme, which is needed for the drug metabolism into an active metabolite; in case of co‐administration, it can lead to an increased risk of cardiovascular events and failure of therapy due to this enzyme suppression [4]. It is worthy to mention that clopidogrel is a prodrug, and its active metabolite formation depends greatly on the CYP450 2C19 activities; 2C19 has multiple genotypes which classify into rapid or poor metabolizers and others [5]. In addition to this, approximately 85% of the drug is inactivated after metabolism, so these factors also affect the therapeutic efficacy of clopidogrel [6]. Jute mallow juice consumption with ciprofloxacin can also cause decreased efficacy of the drug as it increases the viscosity in gastric media; whenever the concentration of jute mallow increases, this further delays dissolution and absorption [7]. Complex formation and chelation effect can also cause reduced absorption of certain medications. The absorption of some antibiotics like tetracyclines is also decreased when they are co‐administered with calcium‐rich products due to the chelation effect [8].

In this research drug‐food interaction is studied between spinach ( *Spinacia oleracea* ) which is consumed in many countries around the world, including European countries, where it's consumed as soup or added to green smoothies and Middle East countries where it's cooked as a main meal. It is highly nutritious and rich in metals like manganese, copper, calcium, folate, Vitamin K, and Vitamin A [9].

Clopidogrel is an anticoagulant; it is indicated for the treatment of cardiovascular diseases and prophylaxis from cardiovascular events in high‐risk patients [10, 11]. According to its structure, it has high electronegative atoms including two oxygen atoms, chloride, sulfur, and nitrogen with a CAS RN (120202‐66‐6) [11, 12]. This increases the risk of forming a complex with metals in spinach, as it is rich in positively charged elements magnesium, copper, and iron, which may influence clopidogrel's therapeutic effect. Therefore, this study is of interest for exploring potential interactions between clopidogrel and spinach that could influence drug dissolution under in vitro conditions.

It is expected that ingestion of spinach initially increases gastric pH from (1–2) to (4–6) due to the buffering capacity of the meal, followed by stimulation of gastric acid secretion that gradually restores gastric pH [13, 14]. Ingestion of spinach is expected to increase gastric content viscosity from fasted values (1–2 cP) to approximately (**10–100 cP**), due to swelling of dietary fibers, particularly soluble polysaccharides, which results in a more viscous fed‐state environment. As gastric secretion and digestion process continues, viscosity decreases but remains higher than in the fasted state [13, 15, 16]. The effect of media viscosity on drug disintegration and dissolution occurs due to the decreased wetting and penetration of media liquid into the tablet, which further delays drug disintegration and dissolution, decreasing active pharmaceutical ingredient (API) release and diminishing therapeutic efficacy. Referring to other studies, dissolution of Paracetamol immediate‐release tablet was performed in different media containing a viscosity‐enhancing agent, which is Hydroxypropyl methylcellulose K4M (HPMC K4M). The high viscosity medium was considered to simulate the fed state, and the simple medium without HPMC was considered as the fasting state. Decreased dissolution rates were observed in the higher viscosity medium [17]. Dissolution and absorption using in vitro dissolution absorption system (IDAS2) was performed for multiple drugs including Minoxidil, Carbamazepine, simvastatin, propranolol, acetazolamide, and saquinavir in both fasted and fed state. It was concluded that drugs in Biopharmaceutical classification system (BCS) class I, Minoxidil and propranolol (with high solubility and permeability) are the least affected in high viscosity media, while BCS class II drugs carbamazepine and simvastatin (low solubility and high permeability), dissolution and absorption were the most reduced. Moreover, BCS class IV drugs (low solubility and low permeability) acetazolamide and saquinavir dissolution rates were slowed down, but absorption was not affected [18]. In addition, another study on poor‐permeability drugs Atenolol, metformin, and furosemide showed that an increase in viscosity using HPMC to simulate fasted state resulted in decreased disintegration, dissolution, and absorption of these three drugs, without affecting drugs with pH independent absorption. It was also concluded that different drug formulations could be affected by medium viscosity and further affect drug absorption [19]. To sum up, the pharmacokinetics of the drug is affected by the drug disintegration and dissolution. However, disintegration and dissolution are affected by the drug's physical characteristics, mainly solubility.

This study was aimed to investigate whether spinach could affect the disintegration and dissolution of clopidogrel tablets in vitro, as well as to assess the effects of media characteristics, such as film precipitation and pH. Spinach was selected as a test media since the leaves of this plant are rich in dietary fibers, vitamins, and metals that upon intake may elevate the gastric fluid viscosity. Therefore, the effect of the viscosity and pH of this extract on tablet disintegration and drug release was examined, aiming to find a conclusion on the appropriate administration of this drug concomitantly with spinach. Disintegration and dissolution of clopidogrel tablets were tested in both HCl and phosphate buffer, comparing them to the results in 5% and 7.5% spinach in the mentioned buffers.

## 2 Materials and Methods

### 2.1 Materials

Clopidogrel bisulfate immediate release (IR) 75 mg tablets (Jerusalem Company‐Palestine), Pharma test dissolution apparatus (Hainburg—Germany), Pharma test disintegration apparatus (Hainburg, Germany), Hanna pH meter (Rhode Island, USA), Thermoscientific magnetic stirrer, UV spectrophotometer Unico (NJ, USA), HPLC Agilent 1200 series by Merck Pharmaceutical company (NJ, USA). Analytical grade acetonitrile and phosphoric acid for mobile phase were purchased from Sigma Aldrich (St. Louis, USA). Purified water delivered by thermo scientific system (Waltham, USA). Potassium chloride, hydrochloric acid, sodium hydroxide, and potassium dihydrogen phosphate were purchased from Sigma Aldrich (St. Louis, USA). Brookfield Viscometer (MS, USA), and *Spinacia oleracea* leaves were bought from a local vegetables and fruit store in Ramallah.

### 2.2 Methods

#### 2.2.1 Study Media

USP hydrochloric acid buffer pH was adjusted to 2 and prepared by adding 4.45 g of Potassium chloride (KCl) and 1.983 mL of 37% HCl in 1000 mL purified water (0.1 N Hydrochloric acid), which simulates gastric media. Use of spinach plant as well as its collection in this research complies with relevant institutional, national and international guidelines and legislation.

Phosphate buffer pH was adjusted to 6.8 and prepared by dissolving 6.8 g of potassium dihydrogen phosphate KH_2_PO_4_ 0.897 g Sodium Hydroxide (NaOH) in 1000 mL purified water, which simulates intestinal media. pH was measured in triplicate for each media using a Hanna pH meter.

Spinach media was prepared in two different concentrations (5% and 7.5%) in both buffers. The fresh leaves of spinach were cut, weighed based on the concentration desired, 50 and 75 g respectively. Then, they were added to 1000 mL of buffer and boiled for 10 min at 100C. Disintegration was performed, and then the media was sieved using a 250 μm sieve mesh size to remove all leaves from the media before starting the dissolution process.

No independent adjustment of ionic strength, osmolarity, or buffer capacity was performed following the addition of spinach extract, as such changes are intrinsic to food–drug co‐exposure and were therefore considered part of the experimental condition rather than a confounding variable.

Spinach extract was prepared and used as a whole food–derived matrix without further compositional characterization. Detailed analysis of metal ion content or fiber composition was not performed, as the objective of the study was to assess the net effect of the intact dietary matrix on clopidogrel release rather than to attribute effects to individual components. The pH of the dissolution media was controlled by the buffer systems used, and any intrinsic properties of the extract were therefore evaluated within these standardized dissolution conditions.

#### 2.2.2 Viscosity Measurement

Rheology was measured for the buffers with and without spinach using a Brookfield viscometer with a T‐A #91 spindle. All measurements were conducted in triplicate at temperature 37°C and 100 rpm shear rate.

#### 2.2.3 Disintegration Study

Tablet disintegration tests were performed using a USP disintegrator apparatus (Pharma test DIST‐3); the basket rack assembly consisted of six open‐ended transparent tubes, each tube was provided with a cylindrical disk. Media used for disintegration was HCl buffer, phosphate buffer, 5% and 7.5% spinach in both buffers. The tests were carried out at temperature 37°C in 800 mL solution, using six tablets for each test.

#### 2.2.4 Drug Release Study

Dissolution tests were performed in a USP II paddle apparatus (Pharma test) in HCl buffer, phosphate buffer, 5% and 7.5% spinach in both buffers. All experiments were performed in 900 mL solution at 37°C with the paddle stirring speed at 50 rpm. Three mL samples were withdrawn at predetermined time intervals 5, 10, 15, 20, 30, 45, 60, 90, and 120 min. Samples were filtered using a 0.45 μm membrane, properly diluted, and analyzed using HPLC. Following analysis, dissolution versus time profiles in different media were constructed. All concentrations were run 3 times on the HPLC.

#### 2.2.5 UV Analysis

A blank of the pure solvent (buffer) was used to measure the amount of light transmitted through the sample. UV spectrophotometer by Unico was used to determine the maximum wavelength of clopidogrel; it has a spectral bandpass of 4 nm and wavelength accuracy of ±2 nm. The wavelength range used in test was 200–1000 nm. The wavelength measured for clopidogrel was 220 nm. UV spectrophotometry was used only for determination of the maximum absorbance wavelength (λmax), while quantitative determination of clopidogrel concentration was carried out exclusively by HPLC.

#### 2.2.6 HPLC Analysis

HPLC Agilent 1200 series was used for analysis of all samples. The system was equipped with a pump (model G1312A), an auto sampler (ALS) (model G1329A), and a Hypersil Gold thermo scientific C18 (250 cm × 4.6 mm), 5 μm column (Paisley, UK), and the detector consisted of UV/VIS operated at 220 nm. Chemstation Software (VersionRev B.04.03 (16)) was used for data processing and evaluation. Mobile phase used was 40% acetonitrile with 60% buffer (10 mL of Phosphoric acid H_3_PO_4_ to 1000 mL water H_2_O making 0.147 M H_3_PO_4_). Prior to use, the mobile phase was filtered through 0.45 μm membrane and degassed by sonication for 10 min. The operating temperature of the column was set at 35°C. The injection volume was 5 μL, and the flow rate was maintained at 1.0 mL/min. The run time was 8 min with a retention time of about 4.6 min.

#### 2.2.7 Sample and Standard Preparations

Sample was prepared by adding one tablet of clopidogrel 75 mg (98 mg clopidogrel bisulfate) in each of the six vessels containing 900 mL buffer at 37°C. Sample concentration was 0.1089 mg/mL clopidogrel bisulfate.

Standard solution was prepared by adding 108.9 mg pure clopidogrel bisulfate and dissolved in 100 mL volumetric flask with methanol. Solution was sonicated for a few minutes, then made up to volume with methanol. Afterwards, 5 mL of stock solution were diluted to 50 mL in a volumetric flask with dissolution buffer. 2 mL were withdrawn and serial dilutions were made with 0.1 N HCl until standard solution concentration had reached 0.1089 mg/mL. The use and preparation of *Spinacia oleracea* media was compliant with ethical guidelines and standard practice.

#### 2.2.8 Drug Release and Statistical Analysis

Dissolution results in hydrochloric acid pH 2 simulated gastric fluid—HCl buffer (SGF), and phosphate buffer pH 6.8 simulated intestinal fluid—phosphate buffer (SIF) were compared to dissolution results of 5% and 7.5% spinach in both buffers. Comparison was done using difference and similarity factors *f*_1_ and *f*_2_, respectively.

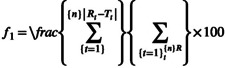

f_2=50\log├├1+\frac1n∑_t=1^nR_t−T_t^2┤^−0.5×100┤

where *R*_*t*_ is considered the amount of drug dissolved in reference solution as in HCl buffer and Phosphate buffer. *T*_*t*_ is considered the amount of drug dissolved in test solution as in 5% and 7.5% spinach in both buffers. An *f*_2_ of higher than 50 indicates that amounts dissolved in reference and test solutions are very similar. Where if *f*_1_ is less than 15, this indicates a difference between amounts dissolved in reference and test solutions.

Dissolution efficiency (DE) is calculated to determine the amount of drug released during the dissolution process minutes compared to maximum release after dissolution is over. Where *T* = 120mins, *y*_100_ is the maximum dissolution and *y*_*t*_ is the percentage of drug dissolved from time zero up to time T. The numerator is equal to AUC from time 0 to time T [20].

$$DE_{T}=\backslash \text{frac}\left\{\int_{0}^{T}y_{t},\mathrm{dt}\right\}\left\{y_{\left\{100\right\}}\cdot T\right\}$$

AUC: Represents the area under the curve, ∑*i* = *n* i = 1: This symbol represents the summation from *i* = 1–*n*, meaning you're adding up the areas of the trapezoids for all points in your data, *t*_1_–*t*_*i*−1_: This represents the width of each trapezoid, *t*_*i*−1_ is the *x*‐value of the previous point, and *t*_1_ is the *x*‐value of the current point (*y*_i−1_ + *y*_*i*_): This represents the average *y*‐value (the height) of each trapezoid, *y*_i−1_ is the *y*‐value of the previous point, and *y*_*i*_ is the *y*‐value of the current point, 1/2: This factor is part of the trapezoid area formula (1/2 × base × height) [21].

$$\mathrm{AUC}=\sum_{i=1}^{i=n}\left(t_{1}-t_{i-1}\right)\times \left(y_{i-1}+y_{i}\right)/2$$

#### 2.2.9 GastroPlus Analysis

GastroPlus software (version 9.8, Simulations Plus Inc., Lancaster, CA, USA), which is based on the Advanced Compartmental Absorption and Transit (ACAT) model, was used for performing IVIVE in phosphate and HCl buffer. GastroPlus software is a physiologically based software program that simulates drug absorption, pharmacokinetics, and pharmacodynamics for several dosage forms in humans and animals. The required input parameters for clopidogrel bisulfate and pharmacokinetic factors either were determined experimentally or were obtained from the literature. These parameters are mentioned in Table 1. The in vivo pharmacokinetic profile of the reference product clopidogrel bisulfate was obtained from Jerusalem Company‐Palestine bioequivalence profile, where Jordan River Pharmaceutical Industries‐Jordan sponsored the study.

**TABLE 1:** Clopidogrel bisulfate parameters inputted into GastroPlus software.

Parameter	Parameter value
Molecular weight (g/mol)^a^	419.9
Solubility at pH 1.2 (mg/mL)	40
Solubility at pH 3.5 (mg/mL)	1.79
Solubility at pH 4 (mg/mL)	0.279
Solubility at pH 4.5 (mg/mL)	0.171
Solubility at pH 5.12 (mg/mL)	0.083
Solubility at pH 6.6 (mg/mL)	0.082
Solubility at pH 6.8 (mg/mL)^a^	0.079
LogP (pH 7)^a^	4.03
Dose (mg)^b^	75
Pka^a^	4.55
Peff (Proximal jejenum in rats) (cm/s × 10^4^)^a^	28.5
Body weight^e^ (kg)	72
Volume of distribution (L) (V_c_)^c^	631.9
Clearance (L/h)^c^	16 980
Unbound percent in plasma^a^	2%
K_12 fasted_ ^c^	0.518
K_21 fasted_ ^c^	0.268
Formulation^b^	Tablet
Dose volume^e^ (mL)	240
Simulation time (h)^d^	24
T_1/2_ ^c^ (h)	7.38
Diffusion coefficient^d^ (cm × 10^2^/×10^5^)	0.75
Mean precipitation time (s)^d^	900
AUC_inf_ (ng.h/mL)^e^	7.708

^a^ Taken from references [22, 23, 24, 25, 26, 27].

^b^ Experimental parameter.

^c^ Value calculated by GastroPlus.

^d^ GastroPlus standard.

^e^ Jerusalem Company.

#### 2.2.10 Study Design

In vitro disintegration and dissolution tests of clopidogrel tablets in hydrochloric acid and phosphate buffer were performed. Furthermore, the effect of the addition of spinach to the mentioned buffers at different concentrations was evaluated. Viscosity and pH measurement for all media at 37°C temperature was done to study the effect of changing these two factors on the disintegration and dissolution of clopidogrel.

Disintegration and dissolution experiments were designed to address different aspects of the clopidogrel–spinach interaction and were therefore conducted under intentionally distinct conditions. Spinach leaves were retained during disintegration testing to evaluate surface‐related effects, including film precipitation and physical interactions that may influence tablet breakup. In contrast, dissolution testing was performed using the USP II paddle apparatus, which necessitates a homogeneous medium to maintain consistent hydrodynamic conditions. As a result, spinach leaves were removed and a clarified spinach extract was used for dissolution studies. This approach enabled evaluation of solution‐mediated effects, including changes in pH and potential metal–drug complexation, while avoiding mechanical disruption of the dissolution process. The use of different media compositions represents a limitation of the study and should be considered when comparing disintegration and dissolution outcomes.

## 3 Results

### 3.1 Viscosity and pH Measurements

**FIGURE 1:**
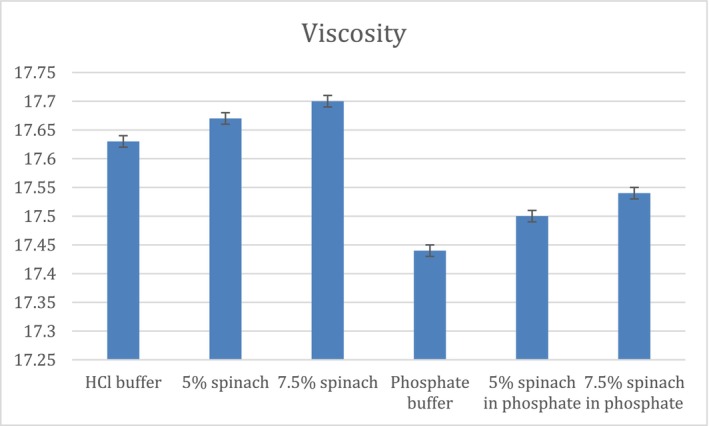
Rheological measurements of all media at 100 rpm.

**FIGURE 2:**
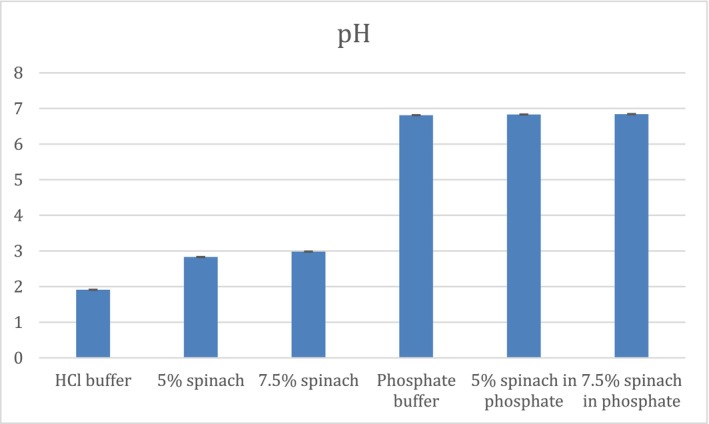
pH measurements of all media.

The viscosities and pH of all studied media measured in triplicate are shown in Figures 1 and 2, respectively. The viscosity measurements showed a minimal increase with the addition of a higher concentration of spinach. Viscosity increase was 17.63, 17.67 to 17.7 cP respectively, in HCl buffer, and 17.44, 17.5 to 17.54 respectively, in phosphate buffer. The viscosity of the media following spinach addition ranged from 17.44 to 17.54 cP and 17.63 to 17.7 cP. These values fall at the lower end of the reported fed‐state gastric viscosity range. The differences between conditions were minimal and within a narrow range. On the other hand, the addition of spinach slightly increased the pH of the HCl buffer 1.91, 2.83 to 2.98 respectively, as spinach extract concentration increased. Which had an impact on the dissolution of clopidogrel, whereas the pH of phosphate buffer increased from 6.81 to 6.83 and 6.84, standard deviation for all pH and viscosity values was 0.01.

### 3.2 Disintegration

**FIGURE 3:**
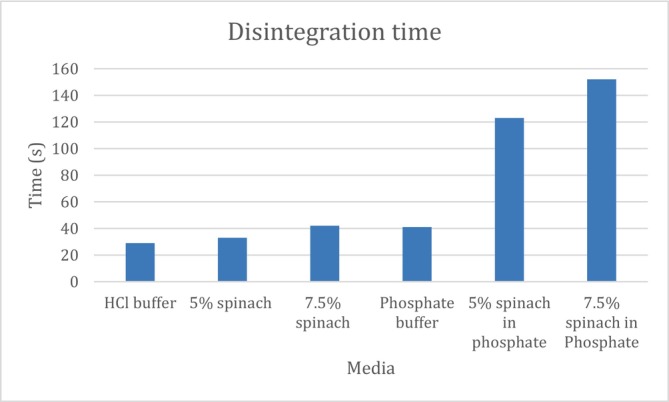
Disintegration time in SGF and SIF, 5% and 7.5% spinach in SIF and SGF with spinach leaves at 37°C.

As seen in Figure 3, disintegration time increased as the concentration of spinach increased due to the addition of spinach leaves to the media; this directly correlates with the minimal increase in viscosity, as well as increased leaf precipitation on tablet surface. Disintegration time in phosphate buffer, 5% spinach, and 7.5% spinach were 41 s, 123 s, and 152 s, respectively. However, in HCl buffer it was 29 s, 33 s, and 42 s, respectively.

### 3.3 Dissolution

**FIGURE 4:**
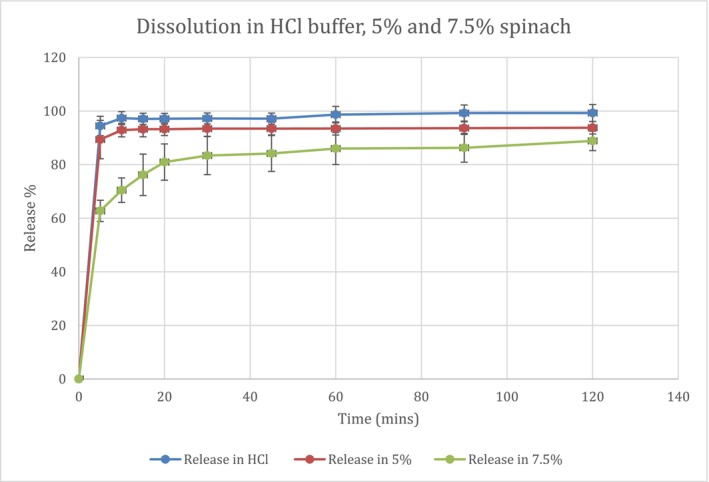
Dissolution curve of clopidogrel tablets in three different media, HCl buffer, 5% spinach and 7.5% spinach in HCl buffer, for clarity error bars represent standard deviation.

**FIGURE 5:**
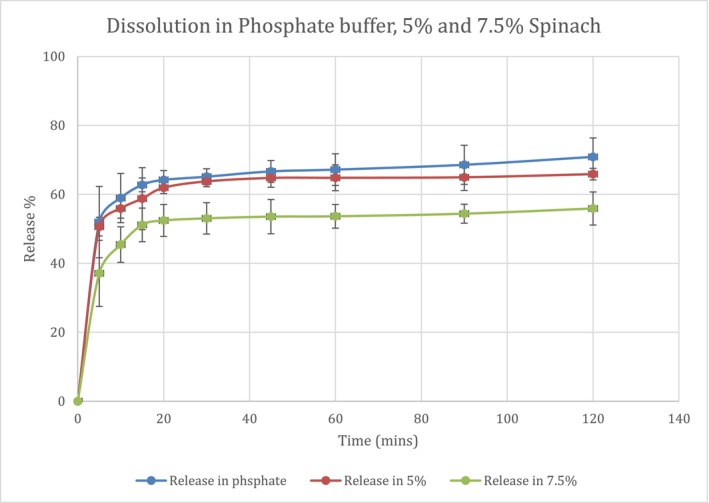
Dissolution curve of clopidogrel tablets in three different media, phosphate buffer, 5% spinach and 7.5% spinach in phosphate buffer, for clarity error bars represent standard deviation.

The release of clopidogrel bisulfate 75 mg tablets in various buffer media is presented in Figures 4 and 5. Dissolution was significantly reduced as spinach concentration increased from 5% to 7.5% in both HCl buffer (*p*‐value = 0.037) and phosphate buffer (*p*‐value = 0.003), based on ANOVA and Tukey post hoc tests. The similarity factors were different for the dissolution in HCl buffer with 5% and 7.5% spinach (*f*_2_ 65% and 35.8% respectively), when compared to HCl buffer. This indicates a significant difference in the dissolution of clopidogrel, mainly in 7.5% spinach in HCl buffer compared to HCl buffer (*p*‐value < 0.001). As well as the *f*_2_ value, it was 67% and 34.9%, similarity factor result comparing 5% spinach in phosphate buffer to buffer, and 7.5% spinach in phosphate buffer to buffer, respectively. It is noticeable that clopidogrel tablets showed a lower release in phosphate buffer compared to HCl buffer in all media, as shown in Figure 4, which is supported by literature, and this is further discussed in the discussion section. Dissolution efficiency (DE%) in HCl was 94.42%, 88.12% and 80.49% in HCl buffer, 5% spinach and 7.5% spinach, respectively. Moreover, in phosphate buffer DE had the following values, 63.88%, 61.07% and 50.94% in phosphate buffer, 5% spinach and 7.5% spinach, respectively. These values indicate reliable dissolution results.

Analysis of variance (ANOVA) test was done followed by Tukey as post hoc test, which revealed distinct effects of spinach extract concentration on clopidogrel release depending on the dissolution medium. As shown in Table 3, clopidogrel release in HCl buffer was significantly reduced in the presence of both 5% (*p*‐value = 0.019) and 7.5% spinach extract (*p*‐value < 0.001) compared with buffer. Additionally, release in 7.5% spinach extract was significantly lower than in 5% spinach extract (*p*‐value = 0.037), as mentioned in Table 2. In contrast, in phosphate buffer, no statistically significant difference was observed between buffer and 5% spinach extract (*p*‐value = 0.145), whereas release in 7.5% spinach extract was significantly lower than both phosphate buffer (*p*‐value < 0.001) and 5% spinach extract (*p*‐value = 0.003).

**TABLE 2:** Statistical analysis across HCl buffer, 5% and 7.5% spinach media.

Comparison	Media 1 (mean ± SD)	Media 2 (mean ± SD)	*p*
Buffer vs. 5% spinach	Buffer: 99.28 ± 3.16	5% spinach: 93.77 ± 2.33	0.019
Buffer vs. 7.5% spinach	Buffer: 99.28 ± 3.16	7.5% spinach: 88.86 ± 3.63	< 0.001
5% vs. 7.5% spinach	5% spinach: 93.77 ± 2.33	7.5% spinach: 88.86 ± 3.63	0.037

### 3.4 Gastroplus

**FIGURE 6:**
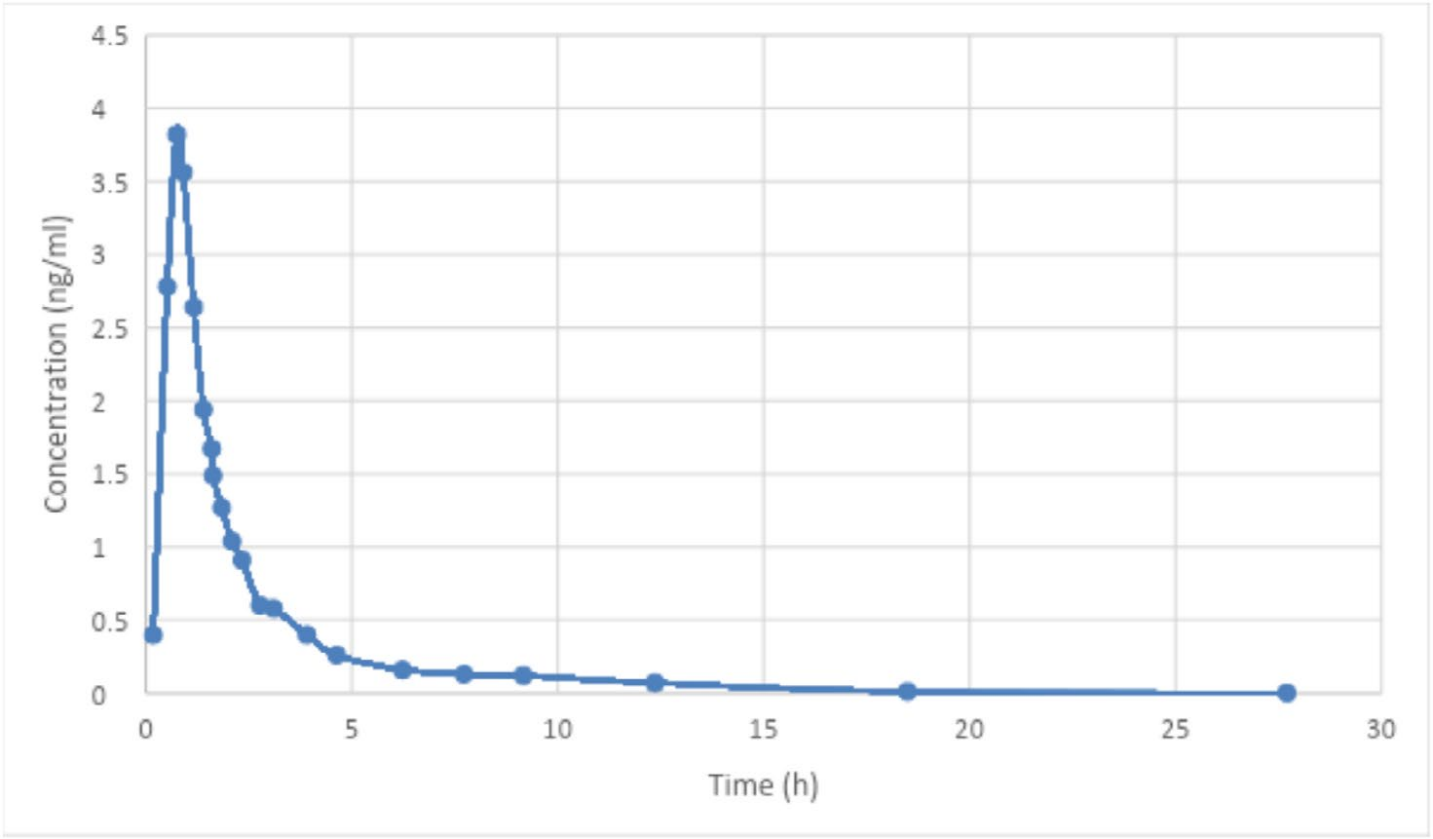
In vivo plasma concentration of clopidogrel versus time (by Jerusalem Company).

When the in vitro outcomes obtained in this study were compared with the numbers in Figure 6. Results have shown that there's weak in silico and in vivo correlation between the in vitro results in HCl buffer, and in vivo results obtained from Jerusalem Company for the drug clopidogrel, as seen in Table 4. In HCl buffer, *R* ^2^ value equaled 0.736692, compared to IVIVE with phosphate buffer, where *R* ^2^ value was 0.693233.

## 4 Discussion

*Spinacia oleracea* was used as a media to study its effect on film precipitation, pH and dissolution of clopidogrel, as spinach is a very popular food consumed worldwide in smoothies, salads and main meals, because it is highly nutritious and can be grown in many regions worldwide. The choice of 5% and 7.5% spinach concentration was based on regular human dietary intake, which is ½–1 cup of cooked spinach or 2–3 cups raw spinach. Spinach leaves were boiled to prepare an aqueous extract suitable for in vitro disintegration and dissolution experiments. This approach was implemented to ensure a homogeneous test medium compatible with the USP II paddle apparatus and to isolate the effects of dissolved spinach constituents on clopidogrel performance. Boiling was also intended to simulate common dietary consumption, as spinach is frequently consumed in cooked forms such as soups. While boiling may alter the native composition of spinach, this methodology enabled focused evaluation of solution‐phase interactions, including pH changes, potential metal complexation and film precipitation. However, this should be considered when interpreting the results.

Disintegration time was shorter in HCl and phosphate buffer media than in spinach extract media, which may be attributed to the coating effect of spinach leaves on the tablet surface, leading to delayed disintegration. Nevertheless, disintegration occurred within 5 min in all media, indicating that this test alone is not sufficient to make a conclusion on the drug's in vitro performance. The prolonged disintegration observed in 5% and 7.5% spinach media is mainly associated with surface coverage by spinach leaves that reduced the area exposed to the medium. Together, these factors likely hindered fluid penetration into the tablet matrix, thereby delaying disintegration. Disintegration studies were conducted in simulated intestinal fluid and compared with 5% and 7.5% spinach media without leaf removal, in order to assess the impact of film precipitation on fluid penetration.

An increase in medium viscosity is known to reduce fluid penetration into tablets and prolong disintegration time. Similar effects have been reported in studies investigating negative food effects, where the presence of food in the gastrointestinal tract reduced drug availability [19]. However, in the present study, the measured increases in viscosity were minimal and therefore unlikely to be the primary contributor to the observed delay in disintegration. Instead, film precipitation on the tablet surface appears to play a more dominant role. This is supported by the comparison between disintegration in SIF and in SIF containing 5% spinach, where the presence of spinach delayed disintegration by 1.37 min despite only minor changes in viscosity. Referring to Figure 1, although viscosity showed a gradual increase with increasing spinach concentration (phosphate buffer 17.44 cP respectively < 5% spinach 17.5 cP < 7.5% spinach 17.54 cP), this increase was limited and may be attributed to higher concentrations of extracted metals resulting from boiling larger amounts of spinach leaves. Although viscosity was measured to characterize the physicochemical properties of the spinach‐containing media, the absolute viscosity values and the minimal differences observed between conditions are unlikely to be physiologically meaningful or to significantly influence tablet disintegration or dissolution. Therefore, viscosity is not considered a primary contributor to the observed effects. The discussion is instead focused on pH‐dependent behavior and film precipitation phenomena, which are more likely to impact drug release under the studied conditions. Viscosity measurements were performed in the same media used for dissolution after removal of spinach leaves to isolate the effects of dissolved components and potential chemical interactions with the drug. Direct viscosity measurement in the presence of spinach leaves was not feasible due to equipment limitations, as the Brookfield viscometer requires homogeneous fluids and suspended leaves could have produced inaccurate readings [26, 28, 29].

Dissolution in both SGF and SIF media had been significantly reduced with the addition of 7.5% *S. oleracea* extract (ANOVA test, *p*‐value < 0.001 in both media) inversely proportional with its concentration. The reduction in dissolution could be explained by pH increase in SGF, as spinach concentration increased from 5% to 7.5%, pH increase has led to decreased dissolution of clopidogrel; this finding is supported by a previous study showing that clopidogrel solubility is reduced at higher pH values [23]. This also complies with dissolution results seen in Figures 4 and 5, release of clopidogrel in phosphate buffer at 120 min was almost 70% compared to 98% in HCl buffer, so this drug has better solubility in a lower pH media. Clopidogrel bisulfate is a weak base with a pKa of 4.55 [25], so in vitro dissolution is reduced by increased pH, which is consistent with other studies on fluconazole and dipyridamole drugs that are also weak bases, whose in vitro and in vivo dissolution decreased at higher pH [30, 31, 32]. Same as other weakly basic drugs from BCS class II, which the drug clopidogrel is classified under, that have lower solubility at higher pH including carvedilol that had been studied formerly [33]. However, bioavailability data indicates that clopidogrel absorption is incomplete < 50%, despite its typical classification as BCS Class II, which reflects limited effective permeability in vivo which is often considered as borderline behavior between BCS II and IV [34]. Referring to Figure 2, pH increase from 1.91 to 2.98 in HCl buffer and 7.5% spinach, respectively, shows a reduction in dissolution of clopidogrel bisulfate from 99.3% to 88.6%, which is justified in previously mentioned literature. In addition to the mild pH increase in phosphate buffer from 6.81 to 6.84, that has also led to reduction of dissolution of clopidogrel from 70.9% to 55.9%, in phosphate buffer and 7.5% spinach in phosphate, respectively. In phosphate buffer, the pronounced reduction in dissolution cannot be attributed solely to the minimal pH increase observed following spinach addition (6.81 to 6.84), which is insufficient to substantially affect the dissolution of a weakly basic drug. Moreover, because spinach leaves were removed from the medium prior to dissolution testing, the observed effects cannot be attributed to direct physical interference by spinach leaves. These findings indicate that pH is not the primary determinant of dissolution behavior in phosphate buffer. Instead, the results are more consistent with solution‐phase phenomena involving solubilized spinach constituents in the presence of phosphate ions, which may impede drug release despite minimal changes in pH.

This can be due to the increase in pH and possible complex formation as well.

Similarity factor (*f*_2_) analysis revealed a significant decrease in the dissolution of clopidogrel tablets in 7.5% spinach medium (*p*‐value < 0.001 in both media). The *f*_2_ value was 35.8% when compared with HCl buffer and 34.9% when compared with phosphate buffer, confirming substantial differences in dissolution behavior. In contrast, the similarity factor comparing HCl buffer with 5% spinach medium was 65%, indicating no significant change in dissolution. In addition, dissolution results for phosphate buffer were comparable to those in HCl buffer, with an *f*_2_ value of 67% when phosphate buffer was compared to 5% spinach medium, reflecting an insignificant difference (ANOVA test, *p*‐value 0.145).

Jerusalem Company provided the in vivo pharmacokinetic profile of the reference clopidogrel product (Clobed, 75 mg as bisulfate) in graphical form only, as additional data were confidential and unavailable. These data originated from a clinical bioequivalence study conducted during generic product development. In vivo concentrations were used for IVIVE analysis in GastroPlus; however, the resulting correlations were modest (*R* ^2^ = 0.74 in HCl buffer and 0.69 in phosphate buffer). Due to the limited description of the in vivo data and the absence of dissolution‐to‐absorption scaling assumptions (e.g., Fa, Fdiss, and precipitation), the IVIVE results are considered exploratory and hypothesis‐generating only and do not support predictive or translational conclusions.

As shown in Tables 2 and 3, statistical analysis of dissolution across all media showed a concentration‐dependent inhibitory effect of spinach extract on clopidogrel release, which was more pronounced at the higher extract concentration of 7.5% in both phosphate and HCl buffer. However, in HCl buffer, there was a statistically significant reduction in dissolution at 5% spinach as well (ANOVA test, *p*‐value 0.019).

**TABLE 3:** Statistical analysis across phosphate buffer, 5% and 7.5% spinach media.

Comparison	Media 1 (mean ± SD)	Media 2 (mean ± SD)	*p*
Buffer vs. 5% spinach	Buffer: 70.88 ± 5.49	5% spinach: 65.88 ± 1.68	0.145
Buffer vs. 7.5% spinach	Buffer: 70.88 ± 5.49	7.5% spinach: 55.90 ± 4.80	< 0.001
5% vs. 7.5% spinach	5% spinach: 65.88 ± 1.68	7.5% spinach: 55.90 ± 4.80	0.003

**TABLE 4:** IVIVE by GastroPlus results.

Media	*R* ^2^ value
HCl buffer	0.736692
Phosphate buffer	0.693233

Based on viscosity, pH and GastroPlus results that have been obtained in this study, it can be concluded that the main cause behind diminished dissolution of clopidogrel in *S. oleracea* extract is due to increased pH at higher spinach concentrations, where fed state usually results in increased gastrointestinal pH as approved by a former study [35] It is not possible to conclude if a metal interaction has occurred between metals extracted from spinach and clopidogrel, but this is highly suggestive as it was explained in the introduction. Further studies including Infrared Spectrometry can be done to study the chemical interaction on the molecular level and determine the cause behind the reduction of in vitro concentration of clopidogrel in spinach media. In addition, the viscosity of the media with spinach leaves can be measured in future studies, as the viscosity could not be measured with leaves in media due to limited available equipment for viscosity measurement.

One of the proposed mechanisms for the interaction between clopidogrel and spinach is the possibility of chelation between metals in spinach extract and clopidogrel. Based on clopidogrel bisulfate chemical structure (added below), it has free ions, so it has chelation potential when added to spinach extract, which is rich in several metals and vitamins. On the other hand, spinach has chelating properties, as it is rich in iron, copper, vitamin C, and vitamin K. In addition, spinach can contain heavy metals including lead, cadmium, and mercury based on levels of these metals in the soil it is grown in [5, 28, 29, 36].

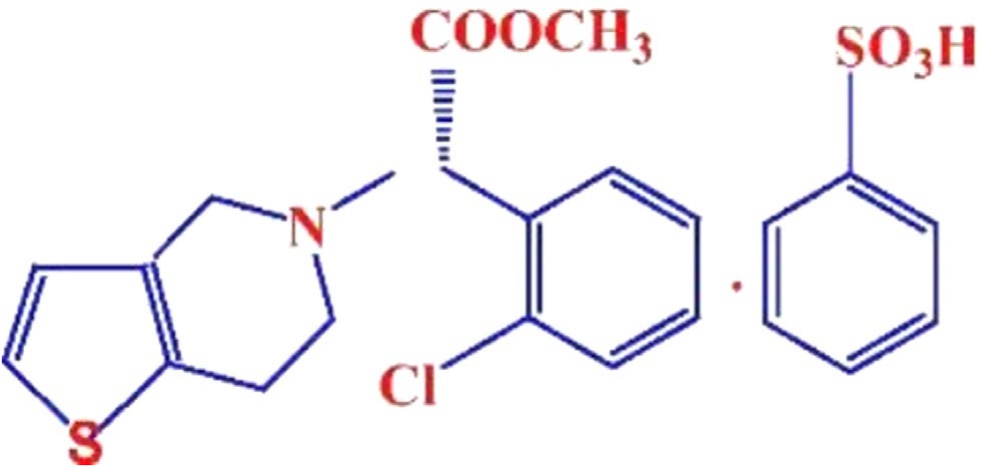

Spinach behavior in the digestive tract was not evaluated, due to the limitation of conducting a clinical trial on humans in our region, in addition to the limitation of materials if this was to be performed in the laboratory. Therefore, this can be done in future research. Studying whether a complex forms between spinach extract and clopidogrel by infrared spectroscopy might be useful. In addition, a simulation of in vitro in vivo correlation studying the behavior of spinach can be done in future studies for more reliable conclusions.

## 5 Conclusion

This study demonstrates the presence of an in vitro interaction between clopidogrel and *Spinacia oleracea* extract, as evidenced by reduced disintegration and dissolution in spinach‐containing media. The observed reduction in dissolution was primarily associated with increased medium pH and film precipitation on the tablet surface, while additional factors such as potential complex formation between clopidogrel and components of the spinach extract may also have contributed. These findings are limited to in vitro experimental conditions and modeling outputs and do not allow direct interpretation of therapeutic impact. Consequently, no dietary recommendations regarding spinach consumption with clopidogrel can be made based on the present results alone. Further investigations, including in vivo evaluations, are required to better characterize the interaction and to determine whether the observed in vitro effects translate into clinically meaningful outcomes.

## Author Contributions

**Dana Sadaqa:** investigation, writing – original draft, methodology, writing – review and editing, software, formal analysis, data curation, validation. **Abdullah K. Rabba:** formal analysis, writing – review and editing. **Asma Radwan:** software, writing – review and editing. **Hani A. Naseef:** supervision, methodology, funding acquisition, writing – review and editing. **Ramzi H. M. Muqedi:** HPLC analysis, writing – review and editing.

## Funding

The authors have nothing to report.

## Ethics Statement

The authors have nothing to report.

## Consent

The authors have nothing to report.

## Conflicts of Interest

The authors declare no conflicts of interest.

## Supporting information


**Data S1:** prp270223‐sup‐0001‐DataS1.xlsx.

**Data S2:** prp270223‐sup‐0002‐DataS2.xlsx.

**Data S3:** prp270223‐sup‐0003‐DataS3.xlsx.

**Data S4:** prp270223‐sup‐0004‐DataS4.xlsx.

## Data Availability

The in vivo data in this study are available from Jerusalem Pharmaceutical Company, but restrictions apply to the availability of these data, which were used under license for the current study, and so are not publicly available. However, excel files for linearity and dissolution profiles were uploaded alongside this manuscript; other data generated or analyzed in this research are included in this published article. Supporting Information supporting this work is available in the Supplementary Section. It provides additional data and details that complement the main text.

## References

[1] F. Omachi , M. Kaneko , R. Iijima , M. Watanabe , and F. Itagaki , “Relationship Between the Effects of Food on the Pharmacokinetics of Oral Antineoplastic Drugs and Their Physicochemical Properties,” Journal of Pharmaceutical Health Care and Sciences 5, no. 1 (2019): 1–8, 10.1186/S40780-019-0155-1. 31827876 PMC6889584

[2] P. G. Welling , “Influence of Food and Diet on Gastrointestinal Drug Absorption: A Review,” Journal of Pharmacokinetics and Biopharmaceutics 5, no. 4 (1977): 291–334, 10.1007/BF01061694. 330836

[3] W. A. Dayyih , I. Al‐Ani , M. Hailat , et al., “Review of Grapefruit Juice‐Drugs Interactions Mediated by Intestinal CYP3A4 Inhibition,” Journal of Applied Pharmaceutical Science 14, no. 5 (2024): 59–68, 10.7324/JAPS.2024.160197.

[4] M. T. Holmberg , A. Tornio , M. Neuvonen , P. J. Neuvonen , J. T. Backman , and M. Niemi , “Grapefruit Juice Inhibits the Metabolic Activation of Clopidogrel,” Clinical Pharmacology and Therapeutics 95, no. 3 (2014): 307–313, 10.1038/CLPT.2013.192. 24067745

[5] A. F. Khorshid , “New Analysis of Clopidogrel Bisulfate in Plavix Tablet and Human Biological Fluids Utilizing Chemically Modified Carbon Paste Sensor,” Arabian Journal of Chemistry 12, no. 7 (2019): 1740–1750, 10.1016/J.ARABJC.2014.08.001.

[6] T. A. Clarke and L. A. Waskell , “The Metabolism of Clopidogrel Is Catalyzed by Human Cytochrome P450 3A and Is Inhibited by Atorvastatin,” Drug Metabolism and Disposition 31, no. 1 (2003): 53–59, 10.1124/DMD.31.1.53. 12485953

[7] A. Radwan , A. N. Zaid , N. Jaradat , and Y. Odeh , “Food Effect: The Combined Effect of Media pH and Viscosity on the Gastrointestinal Absorption of Ciprofloxacin Tablet,” European Journal of Pharmaceutical Sciences 101 (2017): 100–106, 10.1016/J.EJPS.2017.01.030. 28163162

[8] W. Guerra , P. P. Silva‐Caldeira , H. Terenzi , and E. C. Pereira‐Maia , “Impact of Metal Coordination on the Antibiotic and Non‐Antibiotic Activities of Tetracycline‐Based Drugs,” Coordination Chemistry Reviews 327‐328 (2016): 188–199, 10.1016/J.CCR.2016.04.009.

[9] J. L. Roberts and R. Moreau , “Functional Properties of Spinach (*Spinacia oleracea* L.) Phytochemicals and Bioactives,” Food & Function 7, no. 8 (2016): 3337–3353, 10.1039/C6FO00051G. 27353735

[10] Y. J. Zhang , M. P. Li , J. Tang , and X. P. Chen , “Pharmacokinetic and Pharmacodynamic Responses to Clopidogrel: Evidences and Perspectives,” International Journal of Environmental Research and Public Health 14, no. 3 (2017): 301, 10.3390/IJERPH14030301. 28335443 PMC5369137

[11] C. J. Beavers , P. Patel , and I. A. Naqvi , “Clopidogrel,” in Antiplatelet Therapy in Cardiovascular Disease (Wiley‐Blackwell, 2025), 160–165, 10.1002/9781118493984.ch19.

[12] National Center for Biotechnology Information , “PubChem Compound Summary for CID 60606, Clopidogrel,” accessed October 8, 2021, https://pubchem.ncbi.nlm.nih.gov/compound/Clopidogrel#section=LogP.

[13] J. E. Hall , Guyton and Hall Textbook of Medical Physiology (Elsevier, 1946), ClinicalKey, accessed December 25, 2025, https://www.clinicalkey.com/#!/browse/book/3‐s2.0‐C20170004883.

[14] K. E. Barrett , S. M. Barman , H. L. Brooks , and J. X.‐J. Yuan , Ganong's Review of Medical Physiology, 26th ed. (McGraw Hill Medical, 2019), accessed December 25, 2025, https://accessmedicine.mhmedical.com/book.aspx?bookid=2525.

[15] L. Marciani , P. A. Gowland , R. C. Spiller , et al., “Effect of Meal Viscosity and Nutrients on Satiety, Intragastric Dilution, and Emptying Assessed by MRI,” American Journal of Physiology‐Gastrointestinal and Liver Physiology 280, no. 6 (2001): G1229–G1231, 10.1152/AJPGI.2001.280.6.G1227. 11352816

[16] M. Elleuch , D. Bedigian , O. Roiseux , S. Besbes , C. Blecker , and H. Attia , “Dietary Fibre and Fibre‐Rich By‐Products of Food Processing: Characterisation, Technological Functionality and Commercial Applications: A Review,” Food Chemistry 124, no. 2 (2011): 411–421, 10.1016/J.FOODCHEM.2010.06.077.

[17] S. Cvijić , J. Parojčić , and P. Langguth , “Viscosity‐Mediated Negative Food Effect on Oral Absorption of Poorly‐Permeable Drugs With an Absorption Window in the Proximal Intestine: In Vitro Experimental Simulation and Computational Verification,” European Journal of Pharmaceutical Sciences 61, no. 1 (2014): 40–53, 10.1016/J.EJPS.2014.04.008. 24751672

[18] L. D. Simionato , L. Petrone , M. Baldut , S. L. Bonafede , and A. I. Segall , “Comparison Between the Dissolution Profiles of Nine Meloxicam Tablet Brands Commercially Available in Buenos Aires, Argentina,” Saudi Pharmaceutical Journal 26, no. 4 (2018): 578–584, 10.1016/J.JSPS.2018.01.015. 29844730 PMC5961619

[19] A. Radwan , G. L. Amidon , and P. Langguth , “Mechanistic Investigation of Food Effect on Disintegration and Dissolution of BCS Class III Compound Solid Formulations: The Importance of Viscosity,” Biopharmaceutics & Drug Disposition 33, no. 7 (2012): 403–416, 10.1002/BDD.1798. 22782559

[20] S. Dastmalchi , A. Garjani , N. Maleki , et al., “Enhancing Dissolution, Serum Concentrations and Hypoglycemic Effect of Glibenclamide Using Solvent Deposition Technique,” Journal of Pharmacy & Pharmaceutical Sciences: A Publication of the Canadian Society for Pharmaceutical Sciences, Societe canadienne des sciences pharmaceutiques 8, no. 2 (2005): 175–181, https://sites.ualberta.ca/~csps/JPPS8%282%29/M.Barzegar‐Jalali/glibenclamide.htm. 16124928

[21] J. Ye , “General AUC Calculated Based on the Trapezoidal Rule,” Lexjansen.

[22] B. Abrahamsson , T. Albery , A. Eriksson , I. Gustafsson , and M. Sjöberg , “Food Effects on Tablet Disintegration,” European Journal of Pharmaceutical Sciences 22, no. 2–3 (2004): 165–172, 10.1016/J.EJPS.2004.03.004. 15158901

[23] Z. E. Jassim and A. A. Hussein , “Dissolution Method Development and Enhancement of Solubility of Clopidogrel Bisulfate,” International Research Journal of Pharmacy 8, no. 6 (2017): 25–29, 10.7897/2230-8407.08691.

[24] DrugBank , “Clopidogrel: Uses, Interactions, Mechanism of Action,” DrugBank Online, accessed July 22, 2021, https://go.drugbank.com/drugs/DB00758.

[25] CHMP , “Assessment Report Clopidogrel/Acetylsalicylic Acid Teva International Non‐Proprietary Name: Clopidogrel/Acetylsalicylic Acid,” Committee for Medicinal Products for Human Use (CHMP), 2014, www.ema.europa.eu/contact.

[26] T. Incecayir , S. Ilbasmis‐Tamer , F. Tirnaksiz , and T. Degim , “Assessment of the Potential Drug‐Drug Interaction Between Carvedilol and Clopidogrel Mediated Through Intestinal P‐Glycoprotein,” Die Pharmazie 71, no. 8 (2016): 472–477, 10.1691/PH.2016.6059. 29442035

[27] N. A. Farid , A. Kurihara , and S. A. Wrighton , “Prasugrel in Humans Metabolism and Disposition of the Thienopyridine Antiplatelet Drugs Ticlopidine, Clopidogrel, and Prasugrel in Humans,” Journal of Clinical Pharmacology 50 (2010): 126–142, 10.1177/0091270009343005. 19948947

[28] M. Zor and S. Kocaoba , “Determination of Metal Contents in Some Green Leafy Vegetables in Marmara Region of Turkey,” SN Applied Sciences 5 (2023): 154, 10.1007/S42452-023-05369-W.

[29] T. F. Miano , “Nutritional Value of Spinacia Oleraecea Spinach‐An Overview,” International Journal of Life Sciences and Review 2 (2016): 172–174, https://ijlsr.com/bft‐article/nutritional‐value‐of‐spinacia‐oleraecea‐spinach‐an‐overview/?view=fulltext.

[30] N. H. Anderson , M. Bauer , N. Boussac , R. Khan‐Malek , P. Munden , and M. Sardaro , “An Evaluation of Fit Factors and Dissolution Efficiency for the Comparison of In Vitro Dissolution Profiles,” Journal of Pharmaceutical and Biomedical Analysis 17, no. 4–5 (1998): 811–822, 10.1016/S0731-7085(98)00011-9. 9682166

[31] H. Nogami , H. Fukuzawa , and Y. Nakai , “Studies on Tablet Disintegration. I. The Effect of Penetrating Rate on Tablet Disintegration,” Chemical & Pharmaceutical Bulletin (Tokyo) 11, no. 11 (1963): 1389–1398, 10.1248/CPB.11.1389. 14090586

[32] K. Matsui , Y. Tsume , G. E. Amidon , and G. L. Amidon , “In Vitro Dissolution of Fluconazole and Dipyridamole in Gastrointestinal Simulator (GIS), Predicting In Vivo Dissolution and Drug‐Drug Interaction Caused by Acid‐Reducing Agents,” Molecular Pharmaceutics 12, no. 7 (2015): 2418–2428, 10.1021/ACS.MOLPHARMACEUT.5B00135. 25985298

[33] R. Hamed , A. Awadallah , S. Sunoqrot , et al., “pH‐Dependent Solubility and Dissolution Behavior of Carvedilol—Case Example of a Weakly Basic BCS Class II Drug,” AAPS PharmSciTech 17, no. 2 (2015): 418–426, 10.1208/S12249-015-0365-2. 26202065 PMC4984888

[34] M. Javed Qureshi , F. Fuie Phin , and S. Patro , “Enhanced Solubility and Dissolution Rate of Clopidogrel by Nanosuspension: Formulation via High Pressure Homogenization Technique and Optimization Using Box Behnken Design Response Surface Methodology,” Journal of Applied Pharmaceutical Science 7, no. 2 (2017): 106–113, 10.7324/JAPS.2017.70213.

[35] O. Menard , U. Lesmes , C. S. Shani‐Levi , et al., “Static In Vitro Digestion Model Adapted to the General Older Adult Population: An INFOGEST International Consensus,” Food & Function 14, no. 10 (2023): 4569–4582, 10.1039/D3FO00535F. 37099034

[36] A. L. Seyfferth , M. A. Limmer , B. R. K. Runkle , and R. L. Chaney , “Mitigating Toxic Metal Exposure Through Leafy Greens: A Comprehensive Review Contrasting Cadmium and Lead in Spinach,” GeoHealth 8, no. 6 (2024): e2024GH001081, 10.1029/2024GH001081. PMC11181011 38887469

